# Venture from the Interior—Herpesvirus pUL31 Escorts Capsids from Nucleoplasmic Replication Compartments to Sites of Primary Envelopment at the Inner Nuclear Membrane

**DOI:** 10.3390/cells6040046

**Published:** 2017-11-25

**Authors:** Susanne M. Bailer

**Affiliations:** 1 Institute for Interfacial Engineering and Plasma Technology IGVP, University of Stuttgart, Stuttgart 70174, Germany; Susanne.Bailer@igvp.uni-stuttgart.de; Tel.: +49-711-970-4180; 2 Fraunhofer Institute for Interfacial Engineering and Biotechnology IGB, Stuttgart 70569, Germany

**Keywords:** herpesvirus, virus morphogenesis, nuclear envelope, nucleocapsid, nuclear egress, nuclear egress complex NEC, pUL34, pUL31

## Abstract

Herpesviral capsid assembly is initiated in the nucleoplasm of the infected cell. Size constraints require that newly formed viral nucleocapsids leave the nucleus by an evolutionarily conserved vescular transport mechanism called nuclear egress. Mature capsids released from the nucleoplasm are engaged in a membrane-mediated budding process, composed of primary envelopment at the inner nuclear membrane and de-envelopment at the outer nuclear membrane. Once in the cytoplasm, the capsids receive their secondary envelope for maturation into infectious virions. Two viral proteins conserved throughout the herpesvirus family, the integral membrane protein pUL34 and the phosphoprotein pUL31, form the nuclear egress complex required for capsid transport from the infected nucleus to the cytoplasm. Formation of the nuclear egress complex results in budding of membrane vesicles revealing its function as minimal virus-encoded membrane budding and scission machinery. The recent structural analysis unraveled details of the heterodimeric nuclear egress complex and the hexagonal coat it forms at the inside of budding vesicles to drive primary envelopment. With this review, I would like to present the capsid-escort-model where pUL31 associates with capsids in nucleoplasmic replication compartments for escort to sites of primary envelopment thereby coupling capsid maturation and nuclear egress.

## 1. Introduction

The family of Herpesviruses is divided into three subfamilies alpha-, beta-, and gamma-herpesviruses varying in cell tropism, pathogenicity, and the site of latency [1]. Numerous herpesviral species have been identified, of which nine are known to infect humans [2]. Herpes simplex virus types 1 and 2 (HSV1/2), for example, two members of the alpha-herpesviruses, cause recurrent facial and genital lesions, respectively, keratitis or encephalitis. Human cytomegalovirus (HCMV), a beta-herpesvirus, causes mainly symptomless infections in healthy individuals but is the most common cause of congenital infections in humans. Epstein-Barr virus (EBV), a gamma-herpesvirus, causes infectious mononucleosis and is linked to human cancer. Once acquired, all herpesviruses remain associated with their host for the rest of life. Under certain conditions, herpesviruses reactivate and may induce clinically apparent infections potentially threatening their carriers lifelong.

**Figure 1:**
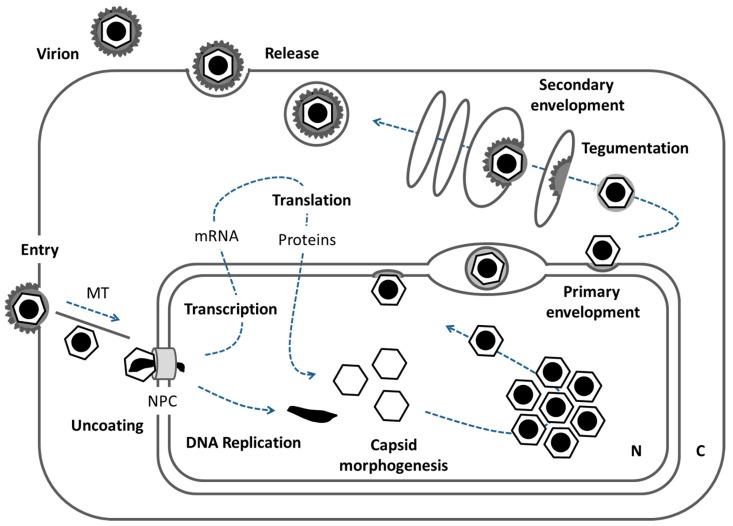
The herpesviral replication cycle. Schematic representation of a host cell and the processes that allow for herpesviral replication. For details see introduction (N: nucleus, C: cytoplasm, MT: microtubules, NPC: nuclear pore complex).

Herpes virions carry a linear double stranded DNA genome, packaged in an icosahedral capsid, which is surrounded by a tegument protein layer, and a host-derived membrane equipped with numerous viral glycoproteins. The herpesviral replication cycle starts upon docking of virions to the host cell followed by entry due to fusion of the virion membrane with the host membrane (Figure 1; [3,4]). Capsids are transported along microtubules (MT) and dock to the cytoplasmic face of the nuclear pore complex (NPC) for uncoating and release of the viral genome into the host nucleus. There, a transcriptional cascade allows for production of viral proteins, furthermore DNA replication of the viral genome proceeds. Morphogenesis of the next generation of herpesviral capsids is initiated in the infected nucleus where a fragile procapsid is packaged with one copy of the viral genome and matured into a stable icosahedral nucleocapsid [5]. In the process of infection, large numbers of nucleocapsids accumulate in replication compartments eventually filling the nucleoplasm and marginalizing the host chromatin [6,7,8], concomitantly, the nuclear lamina is increasingly dissolved [9]. How mature nucleocapsids are released from replication compartments and how they translocate from there to the nuclear envelope is poorly understood [7,10,11,12,13]. In the nuclear periphery, primary envelopment is initiated for vesicular transport of nucleocapsids to the cytoplasm. There, tegumentation and secondary envelopment take place at cytoplasmic membranes resulting in infectious virions released to the extracellular milieu [3,4].

**Figure 2:**
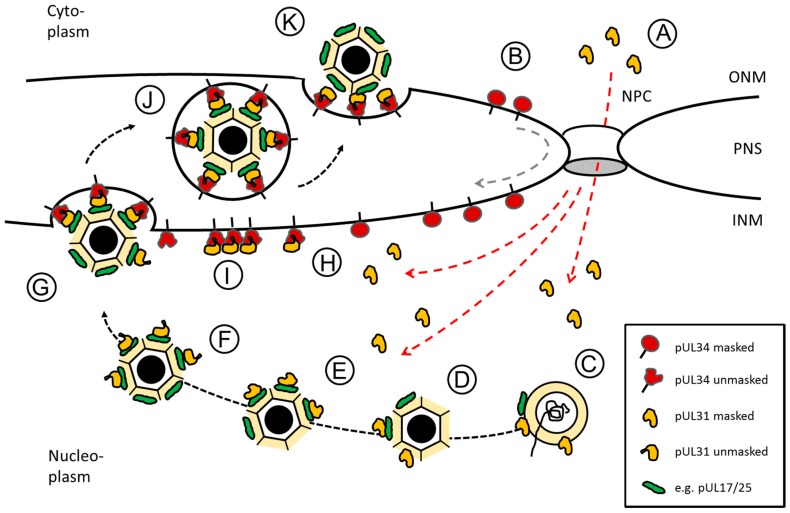
Herpes simplex virus type 1 (HSV1) pUL31-mediated escort of capsids from replication compartments to sites of primary envelopment. Schematic representation of the route, pUL34 and pUL31 take after their synthesis in the cytoplasm until forming the nuclear egress complex (NEC) at the inner nuclear membrane (INM): (**A**) pUL31 (red dashed lines) and (**B**) pUL34 (grey dashed line) are imported into the nucleus independent of each other. pUL34 and pUL31 are masked by conformation and/or unknown binding partners to prevent premature NEC formation. (**C**,**D**) In the nucleus, pUL31 associates with capsids at the sites of their assembly and potentially assists in genome packaging. (**E**) pUL31 recognizes surface components of the capsid e.g., pUL17/pUL25 that mark capsid maturation. (**F**) In presence of the N-terminal domain of pUL31, nucleocapsids are released from replication compartments with pUL31 escorting them to sites of primary envelopment. (**G**) Capsid-associated pUL31 and pUL34 integral to the INM are unmasked to form heterodimeric NECs that assemble into hexagonal lattices to curve around nucleocapsids until primary envelopment is complete. (**H**) NECs or (**I**) NEC seeds preexisting at the INM could contribute to capsid recruitment and envelopment. (**J**) Primary enveloped nucleocapsids temporarily reside in the perinuclear space (PNS). (**K**) Fusion of the primary envelope with the outer nuclear membrane (ONM) allows for release of the nucleocapsids to the cytoplasm for secondary envelopment. The nuclear pore complex (NPC), the outer nuclear membrane (ONM) and the inner nuclear membrane (INM) as well as the perinuclear space (PNS) are depicted (modified after [17]).

Herpesviral nucleocapsids are too large for conventional export through the nuclear pore complex. Instead they escape the nucleus by budding through the nuclear envelope, a process called nuclear egress (Figure 2). Here, nucleocapsids associate with the inner nuclear membrane (INM) and bud into the perinuclear space (PNS) thereby receiving their primary envelope. Upon fusion of the primary envelope with the outer nuclear membrane (ONM), capsids are de-enveloped and released into the cytoplasm for final maturation [4,14]. Previously thought to be exclusive to nuclear egress of herpesviruses, vesicle-mediated export of large ribonucleoprotein particles (RNPs) to the cytoplasm by budding through the inner and outer nuclear membrane was recently described in insect cells [15]. Thus, herpesviruses may exploit a pre-existing and so far unrecognized cellular mechanism for capsid nuclear egress [16].

**Figure 3:**
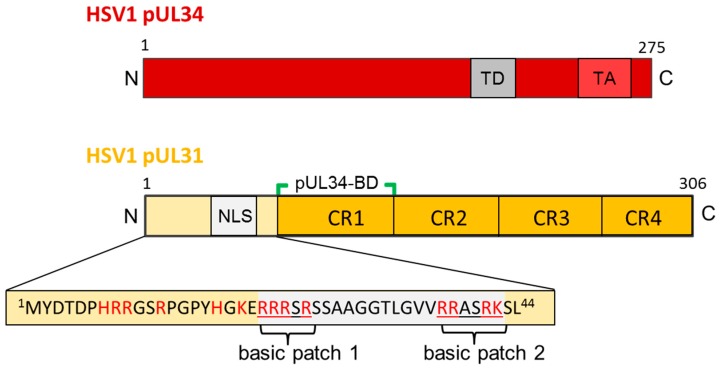
The HSV1 nuclear egress proteins pUL34 and pUL31. Graphical depiction of HSV1 pUL34 and pUL31 and their domain organisation. The amino acid sequence of the N-terminal domain of pUL31 is detailed. N: N-terminus; C: C-terminus; TD: targeting domain; TA: Tail-anchor domain; NLS: nuclear localization sequence; pUL34-BD: pUL34-binding domain; CR: conserved region.

Nuclear egress of all herpesviruses is primarily mediated by two viral proteins, a nucleo-phosphoprotein called pUL31 in HSV1 (Figure 3; [18]), and pUL34, a tail-anchored (TA) type II membrane protein (Figure 3; [19]) as well as their conserved orthologs found throughout the herpesvirus family (HSV1: [19,20]; HSV2: [21,22]; Pseudorabies Virus PrV: [23,24]; Mouse Cytomegalovirus MCMV: [25,26]; HCMV: [27,28]; EBV: [29,30,31]; Kaposi’s sarcoma-associated herpesvirus KSHV: [32,33]). (Unless specified, the nomenclature pUL31 and pUL34 collectively refers to all orthologs.) Together they form the nuclear egress complex (NEC) at the inner nuclear membrane (INM) that is essential for capsid nuclear egress [19,20,23,24,26,28,30,34,35]. The association of pUL34 and pUL31 with primary but not mature virions [24,36,37] is consistent with a dedicated function during primary but not secondary envelopment. 

**Figure 4:**
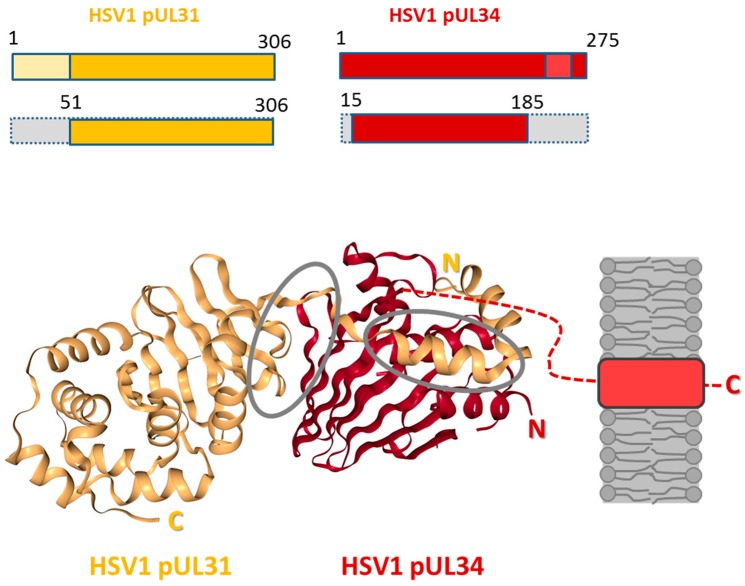
Structure of the HSV1 nuclear egress complex. The structure of the HSV1 pUL31-pUL34 complex is shown as cartoon representation (source: [42]). HSV1 pUL34 and pUL31 and the truncated versions used for the crystals presented are graphically depicted [38]. pUL31 is shown in orange, and pUL34 is shown in red. Interfaces are circled in grey. The inner nuclear membrane (grey) and the tail-anchor domain of pUL34 (light red) are schematically depicted; N: N-terminus; C: C-terminus.

Most recently, the NEC of three different herpesviruses, Herpes simplex virus 1 (HSV1; Figure 4; [38]), Pseudorabies Virus (PrV; [38,39]) and Human Cytomegalovirus (HCMV; [40,41]) has been crystallized. All structures are remarkably similar consistent with nuclear egress being conserved throughout the herpesvirus family.

Isolated co-expression of the two NEC proteins results in empty vesicles that accumulate in the perinuclear space of transfected cells [33,43,44]. In vitro, recombinantly expressed NEC is sufficient to drive membrane budding and scission of intraluminal vesicles into giant unilamellar vesicles [39,45,46]. Consistently, the NEC represents the minimal virus-encoded membrane-budding and scission machinery. This requires a tight spatio-temporal regulation of the NEC activity which likely is achieved by numerous viral and cellular factors present during infection.

The capsid-escort-model proposed in 2015 [17] unites insights of many labs working on various aspects of nuclear egress of herpesviruses. This model envisions a highly orchestrated sequence of events where pUL31 binds to nucleocapsids already in the nucleoplasm for escort to the inner nuclear membrane (INM), subsequent NEC formation and membrane budding (Figure 2), a process potentially supported by NECs preexisting at the INM. Evidence is increasing that this is a common mechanism for herpesviral nuclear egress which allows for coupling of capsid maturation with primary envelopment. 

Taken together, the highly conserved and essential NEC makes nuclear egress an ideal target for pan-herpesviral drugs. Small molecules can now be rationally designed based on extensive structural and mutational data of three different herpesviruses. Compounds could mimic part of the NEC interface with the potential to competitively inhibit NEC formation at the INM. Likewise, compounds could inhibit recruitment of nucleocapsids to the NEC. Either of these antiviral strategies has great potential to affect release of nucleocapsids to the cytoplasm thereby effectively inhibiting viral propagation. 

## 2. The NEC Proteins pUL34 and pUL31 Utilize Separate Routes to Enter the Nucleus

The nuclear envelope is featured by two closely juxtaposed lipid bilayers called outer and inner nuclear membrane (ONM, INM, respectively; Figure 2). Nuclear pore complexes (NPCs) are embedded at sites of membrane fusion to allow for exchange between the cytoplasmic and nuclear compartment. The pore channel is large enough to allow for passive diffusion of soluble proteins smaller than approximately 40 kDa, however most cargos transported between nucleus and cytoplasm use an active transport mechanism mediated by transport factors of the importin β family and a Ran-GTP gradient [47]. In contrast to the well-defined nucleo-cytoplasmic transport of soluble proteins, targeting of integral membrane proteins to the INM is incompletely understood [48,49,50,51]. Integral membrane proteins with cyto-/nucleoplasmic domains smaller than approximately 25 kDa are thought to diffuse along the endoplasmic reticulum (ER) and the peripheral pore membrane and to be retained by interaction with other INM proteins, while proteins with domains larger than 25 kDa may require an active process.

Orthologs of pUL31 are soluble proteins that contain a larger C-terminal domain divided into four conserved regions CR1 to 4 (Figure 2A; [26]). Their N-terminal domain is variable and enriched in basic residues clustered in several patches ([52]; and references therein). Using in silico analysis, putative classical nuclear localization signals (NLS; [52]) have been identified in many but not all pUL31 orthologs and experimentally confirmed upon isolated expression of the respective proteins [17,26,52,53,54,55,56]. In the essential N-terminal domain of HSV1 pUL31, a classical bipartite NLS fulfilled all criteria of a functional NLS (Figure 2A; [17]). However, this NLS was not required for nuclear import of pUL31 in the virus context [17] supporting the existence of a so far unknown redundant import mechanism. Most importantly, the essential function associated with the N-terminal domain of pUL31 that also harbours the NLS, could not be replaced by addition of the SV40-NLS revealing functions other than nuclear import within pUL31-N critical for viral replication. The presence of a nuclear export sequence (NES) within pUL31 is controversially discussed [54,55,57]. While in the PrV ortholog, a NES is debated [54,55], no export activity was found in the HSV1 ortholog [57].

Unlike the soluble pUL31, pUL34 is a tail-anchored (TA) membrane protein (Figure 2B; [17,58]; and references therein). In all pUL34 orthologs, the cyto-/nucleoplasmically exposed N-terminal domains and the C-terminal TA domains are conserved and essential for viral replication (Figure 2B; [58,59,60]). The TA can be replaced by alternative anchor domains [58,60] and deletions of large parts of the linker region between the conserved N-terminal domain and the essential TA domain are tolerated [61]. These data show that the TA of pUL34 primarily serves to membrane-anchor pUL34 and ultimately the NEC. TA membrane proteins represent a specific class of integral membrane proteins characterized by a single transmembrane domain (TMD) positioned at the very C-terminal end. This kind of TMD remains associated with the ribosomal tunnel until translation is complete [62,63,64,65] and requires release from the ribosome for insertion into various target membranes. Posttranslational membrane insertion of pUL34 orthologs occurs in the cytoplasm and thus prior to its targeting to the nucleus [17,52]. While recent progress has been achieved regarding the redundant machinery for posttranslational membrane insertion [66], their intramembrane trafficking in particular to the INM is not completely understood. Interestingly, pUL34 of both PrV and HSV1 contains trafficking signals required to direct these proteins to the INM. In absence of the RQR motif (PrV; [67]) or a multipurpose sequence (HSV1; Funk et al., ms in preparation), pUL34 is mislocalized to the Golgi compartment, consequently, the herpesviral replication is attenuated. Thus, the herpesvirus infection represents an attractive system to study the biogenesis of TA membrane proteins destined to the INM.

Taken together, as shown for the NEC orthologs of HCMV and HSV1, pUL34 and pUL31 utilize separate routes to enter the nucleus (Figure 2A,B; [17,52]). NEC formation in the cytoplasm would prevent trafficking of the NEC partners to the INM thereby compromising their essential nuclear function. Premature interaction could simply be prevented by different expression and/or nuclear import kinetics of the NEC partners. Their high propensity to form the heterodimeric NEC and the budding activity associated with NEC formation [33,43,44,45,68] however suggests that cytoplasmic interaction of the NEC partners is actively inhibited. 

## 3. Association of pUL31 with Capsids in Replication Compartments—A Nuclear Role for pUL31 in Capsid Maturation and Release

In absence of infection, pUL31 targets to the INM and forms the NEC together with the membrane-anchored pUL34 to promote membrane vesiculation [33,43,44]. During infection, formation of empty vesicles is however rarely observed suggesting that also in the nucleus, the NEC activity is tightly controlled. This could be achieved by keeping the NEC that may preexist at the INM, in an inactive form until capsids dock and trigger its budding activity. Alternatively, pUL34 and pUL31 may stay separate and only form the NEC upon recruitment of the nucleocapsid to the INM [69].

Several lines of evidence support a sequence of events where pUL31 associates with nucleocapsids in replication compartments prior to targeting to the INM (Figure 2C–F). During HSV1 infection, wildtype pUL31 locates to nucleoplasmic replication compartments [17], a behavior also observed in absence of pUL34 [20] and particularly pronounced upon deletion or mutation of the N-terminal domain of pUL31 (Figure 2E,F; [17]). The conserved region of pUL31 is sufficient for its targeting to replication compartments; the N-terminal domain however seemed to be functionally important for capsid release [17]: pUL31 that either lacks the complete N-terminal domain (pUL31-ΔN) or two basic patches within this domain is unable to support plaque formation and viral propagation [17,70]. In these mutants, pUL31 could not be detected at the nuclear rim, instead its exclusive nucleoplasmic localization appeared punctuate and correlated with the capsid protein VP5 detected by antibodies to mature hexon epitopes. Mature capsids seemed to be formed at wildtype levels, but remained confined to replication compartments. Replacement of the authentic basic patches with a novel artificial one restored capsid release and translocation to the nuclear envelope, concomitantly viral replication was partially cured. Taken together, while the conserved domains of pUL31 mediate interaction with nucleocapsids, the N-terminal domain is essential for capsid release [70]. 

Strikingly, the interaction of pUL31 with mature capsids already in replication compartments is likely to be conserved in herpesviruses: recent data show, that antibodies recognizing pUL53, the HCMV ortholog of pUL31, specifically decorate mature intranuclear capsids as shown by immunogold labelling of HCMV infected cells (Marschall and Milbradt, personal communication). Importantly, the nucleocapsid periphery was intensely decorated suggesting an efficient association of pUL53 with the nucleocapsid surface in preparation for transport of nucleocapsids to the nuclear periphery and subsequent NEC formation. Proteins that may functionally and/or physically link pUL31 with capsids [4,14] include the minor capsid proteins pUL17 and pUL25 (Figure 2C–F; [69,71,72,73,74]). The heterodimeric pUL17/pUL25 complex associated with the capsid vertex-specific complex (CVSC) is implicated in DNA packaging, capsid maturation and stability ([5,75,76,77,78]; and references therein). Tegument proteins that may associate with capsids already in the nucleus were also suggested to play a role in linking pUL31 and the caspid ([79]; and references therein). Recently, novel candidate interactors of pUL31 were identified using the intra-viral interaction resource for HSV1 HVint [80] and include the capsid protein VP26 encoded by UL35 as well as the protease pUL26 and the glycoprotein gM.

The question arises about the potential role of pUL31 in replication compartments and in association with nucleocapsids. In the nucleoplasm of infected cells, several intermediates of capsid assembly are distinguished, A capsids that lack a viral genome and scaffold, B capsids that are nonproductive intermediates, and C capsids that represent mature capsids with a packaged genome [5]. Preferred nuclear egress of C capsids over A or B capsids was demonstrated suggesting that the surface of mature capsids is marked for selective nuclear egress ([4,14]; and references therein). Production of mature capsids is a complex process involving viral DNA amplification, excision of monomeric genomes and packaging of single genomes into assembled capsids. Several studies report a defect in cleavage/packaging of the viral genome associated with UL31 mutants. The HSV1 UL31 null mutant is associated with reduced viral replication and a defect in genome cleavage/packaging leading to large numbers of empty A and B capsids and only occasional C capsids [34,81]. In EBV, deletion of the pUL31 ortholog BFLF2 results in normal replication and cleavage of the viral genome whereas genome packaging was impaired [31]. A specific dominant negative mutant of MCMV M53 not only blocked nuclear egress but also genome cleavage and capsid maturation [82]. Together, these data suggest a direct role of pUL31 in cleavage and/or packaging of the viral genome (Figure 2C,D; [31,34,82]). In consequence, pUL31 associated with nucleocapsids could mark completion of viral genome cleavage/packaging and allow for selection of encapsidated and mature capsids [69,71,82,83]. Alternatively or in addition, pUL31 could play an indirect role in cleavage/packaging by associating with the pUL17/pUL25 complex thereby stabilizing the complex and the matured capsid (Figure 2E,F; [8,17,34]). 

Little is known how capsids are released from replication compartments [84,85]. Interaction of pUL31 with the matured capsid surface seems to be critical since capsid release is blocked or inhibited in absence or upon mutagenesis of the N-terminal domain of pUL31 [17]. Interestingly, artificial basic residues inserted in the otherwise neutralized N-terminal domain restored capsid release indicating a critical role of the positive charges [17]. Potentially, the N-terminal domain of pUL31 recruits other proteins to modify the capsid environment. A positively charged pUL31 coat could also simply repel the capsids from each other thereby releasing them. The viral kinase pUS3 able to phosphorylate the N-terminal domain of pUL31 [70] may play a regulatory role. However, this is unlikely of great importance, since neither pUS3 nor the phospho-acceptor sites within pUL31-N are essential [70]. Taken together, while the conserved domains of pUL31 mediate interaction of pUL31 with nucleocapsids, the N-terminal domain with its positive charges is essential for capsid release [17]. This scenario would not only coordinate capsid maturation and nuclear egress but at the same time limit premature NEC activity at the INM.

## 4. Nucleocapsids Traverse the Chromatin and the Lamina to Reach the Sites of Primary Envelopment

Capsids assembled and matured in the nuclear interior need to reach the nuclear periphery for primary envelopment and egress to the cytoplasm where they receive their final envelope. In particular during early phases of infection, the densely packed chromatin represents a major barrier. As viral infection progresses, the nuclear architecture is dramatically changed [8,86]. Viral replication compartments increasingly occupy the nuclear space [8]. Concomitantly, the host chromatin is marginalized and the nuclear lamina lining the INM is partially/locally disintegrated to allow for NEC-induced budding. Moreover, infected nuclei significantly increase in size. Nuclei infected with HSV1 mutants lacking either pUL31 or pUL34, however, remain unaltered in chromatin density and size [8], suggesting that both NEC components contribute to intranuclear alterations. Interestingly, several host proteins were identified with potential roles in nuclear expansion that at the same time physically interact with the NEC proteins (Bailer et al., unpublished data). In future, they may provide insight into the underlying molecular processes. 

Initial reports suggested that capsids use the nuclear acto-myosin system for directed movement from replication compartments to the nuclear periphery [10,11,87]. This transport mode is now discussed controversially; instead an actin-independent process was proposed [13,84,86]. Recent reports indicate that capsids can efficiently diffuse through enlarged interchromatin domains generated during herpesviral infection [86]. Furthermore, channels induced during HSV1 infection were demonstrated in the marginalized chromatin of the nuclear periphery where capsids could be found [88,89].

Having traversed the chromatin, the nuclear lamina that lines the INM likely represents another obstacle for egressing capsids. The nuclear lamina forms a dense meshwork composed of various filamentous lamins and of integral membrane lamina-associated proteins (LAPs) [90,91], a structure that supports multiple nuclear functions including nuclear shape and size. During herpesviral nuclear egress, the lamina is fenestrated at sites of nucleocapsid docking and budding while large parts of the lamina remain intact [92]. Thus, nuclear integrity seems to be maintained during herpesviral propagation while at the same time, local alterations of the lamina provide access of nucleocapsids to the INM as well as flexibility for membrane budding. During herpesviral infection, several components of the lamina are modified by phosphorylation potentially resulting in partial dissolution of the lamina [93,94]. 

Recruitment of various cellular kinases to the lamina and the NEC has been reported during infection suggesting that cellular kinases play a complex and potentially redundant role during nuclear egress [94,95]. Importantly, all herpesviruses encode a conserved herpesviral protein kinase (CHPK; called pUL13 in HSV1 and PrV [93,94], pUL97 in HCMV [41,95]), that contributes to lamina dissolution and efficient nuclear egress [95,96]. Furthermore, the viral kinase pUS3 specific for alphaherpesviruses, seems to have various effects on herpesviral infection, including phosphorylation of lamina components [97,98,99,100,101]. Interestingly, pUL13 phosphorylates pUS3 [102] and both protein kinases may cooperate to promote release of capsids from replication compartments [103]. 

## 5. The Nuclear Egress Complex

The NEC represents the crucial hub at the INM to execute primary envelopment of nucleocapsids. Several lines of evidence support its multiple roles in binding of nucleocapsids, membrane vesiculation and rearrangement of the nuclear envelope [93,94]. Proteomic data indicate the recruitment of numerous viral and cellular proteins to the NEC suggesting that primary envelopment is a highly complex process [104,105,106,107,108,109].

Previously, mutational analysis revealed details on the interaction and function of the NEC partners of several herpesviruses. A library of charged cluster mutations identified residues in pUL34 involved in recruiting pUL31 and in NEC function [59]. Similarly, mutational analysis of M50/UL50, the CMV orthologs of HSV1 pUL34, revealed an N-terminal region required for interaction with the pUL31 ortholog M53 [35,110,111]. Within pUL31 orthologs, four conserved regions CR1 to 4 can be distinguished (Figure 3; [26,111,112]) with CR1 providing a binding site for the respective pUL34 orthologs [26,112], while additional binding sites were proposed to exist [22,54,67,82,83,113,114,115]. Collectively, these mutational data on pUL34 and pUL31 interaction(s) unveiled several intermediates of nuclear egress involving the INM e.g., docking of capsids at the nucleoplasmic face, formation of a dense membrane patch, initiation of membrane curvature, and membrane wrapping of capsids for release of enveloped capsids into the perinuclear space [74,92,114,115,116,117]. 

In 2015, five crystal structures of three different herpesviral NECs, of Herpes simplex virus 1 (HSV1; Figure 4; [38]), of Pseudorabies Virus (PrV; [38,39]), and of Human Cytomegalovirus (HCMV; [40,41]) were determined. In addition, the structures of the monomeric HCMV UL53 [41] and Mouse Cytomegalovirus M53 (MCMV; [118]) were solved (for recent reviews see [85,93,94,119]). NECs can be formed out of recombinantly expressed N- and C-terminally truncated pUL31 and pUL34 variants, showing that in case of HSV1, pUL31 (residues 51–306) and pUL34 (residues 15–185) are sufficient to form stable complexes (Figure 4; [45]). Importantly, all NEC structures lack the variable N-terminal extension of pUL31 [38,39,40,41], indicating that while functionally important for nuclear egress of capsids [17], this domain is not required for NEC formation. Several features are conserved in all NEC structures [38,39,40,41]: A heterodimer formed between pUL31 and pUL34 or orthologs is elongated where both pUL34 and pUL31 are organized in a globular manner. The core of pUL34 is formed by a β-sandwich fold that is capped by four helices. In pUL31, the core is made of α-helices and β-strands surrounded by additional α-helices. pUL31 binds a zinc ion that is coordinated by three cysteines and one histidine, a structure strictly conserved in all pUL31 orthologs. The structures revealed two interfaces buried in the NEC: CR1 of pUL31 extends like a V-shaped hook to embrace pUL34 confirming that CR1 of pUL31 is crucial for interaction with pUL34 [26]. To accommodate the hook of pUL31, the β-folds of pUL34 are splayed open reminiscent of a taco thereby forming a groove. The second interface is formed between the globular domains of both proteins. All structural data are consistent with the heterodimeric NEC being highly stable. 

In vivo, co-expression of both NEC proteins is sufficient to invoke the formation of empty vesicles in the perinuclear space indicating that no other viral protein is required for this process [33,43,44]. In vitro, a complex formed out of recombinantly expression and purified components is sufficient to drive membrane vesiculation of artificial membranes in absence of any other viral or host protein [45]. Thus, the NEC is sufficient for both membrane deformation and scission consistent with the NEC being a minimal and complete membrane-budding machinery. Membrane-tethered pUL31 can mediate vesicle formation even in the absence of pUL34 suggesting that pUL31 is directly involved in membrane remodeling [46]. Furthermore, NEC induced vesiculation is very rapid and does not require an energy source. High resolution analysis of NEC-induced vesicles formed in vitro and in vivo using cryo-electron microscopy (cryoEM) revealed, that the NEC oligomerizes into hexagonal cores that assemble into a honeycomb-like lattice [45,68]. The lattice is composed of two tightly interconnected layers that together provide an internal 10 nm scaffold to the budding vesicles where the repeating unit presents with an “archway” motif formed by a single hexagon. 

Modelling of the HSV1 and PrV NEC structures into the respective cyroEM lattice revealed that pUL34 forms the membrane-proximal (MP) part of the NEC while pUL31 provides the membrane-distal (MD) part [39,45,68]. Assembly of heterodimeric NECs into hexameric ring-like structures was observed in two of the crystal preparations [38,40]. These plain ordered arrays resemble the hexagonal lattices observed in capsid-free vesicles formed in vitro [45] or in vivo [68]. A curved lattice could be achieved by tilting hexamers relative to each other thereby establishing specific interhexameric interactions [38,39,40,68].

The MD part of the NEC composed of the C-terminal domain of pUL31 is oriented towards the lumen of the coated vesicle suggesting that this part of pUL31 contacts the capsid surface [38,39,45,68]. This is consistent with the C-terminal domain of pUL31 being sufficient for interaction with capsids in replication compartments [17,54,120]. Since pUL31 was shown to efficiently associate with nucleocapsids in replication compartments ([17]; Marschall and Milbradt, personal communication), heterodimeric NEC formation would occur concomitant with capsid recruitment. Since capsids seem to be extensively decorated with pUL31, pUL31 on nucleocapsids may readily form multiple heterodimeric NECs with pUL34 anchored at the INM, thereby creating avidity effects that drive the membrane envelopment. Alternatively, interaction of nucleocapsids with pUL31 could occur with NECs and NEC seeds already pre-existing at the INM (Figure 2H,I; [68]). 

Nucleocapsids with primary envelopes, or intermediates thereof, are rarely observed in the perinuclear space and do not accumulate during wildtype infection. This suggests that both primary envelopment and de-envelopment are rapid processes. HSV1 and PrV mutant strains with defects in the pUS3 kinase accumulate primary enveloped capsids in the perinuclear space of the infected cell [20,36,121,122] and can be exploited for analysis of nuclear egress [123,124]. Analysis of these primary enveloped nucleocapsids revealed a heptameric organization of the NEC heterodimers in difference to the hexameric organisation observed in empty vesicles [38,39,45,68,124]. Whether this difference in NEC organization is due to the lack of pUS3 or indicates a first step of disassembly induced in the perinuclear space in preparation for de-envelopment is currently unclear. Overall, primary virions are 20% smaller than extracellular mature virions most likely due to the absence of tegument proteins and have very few glycoprotein spikes, distinguishing primary and secondary envelopment processes. Overall, the mechanism for how primary enveloped nucleocapsids leave the perinuclear space is largely unresolved. Recent evidence shows that the LINC complex that physically connects nuclear structures to cytoskeletal elements establishing the equidistant INM and ONM [125] needs to be intact for efficient nuclear egress of nucleocapsids, potentially to promote fusion of primary enveloped nucleocapsids with the ONM [126]. A role of viral fusogenic glycoproteins in this process however is debated controversially [4,14].

## 6. A Model of Orchestrated Nuclear Egress of Capsids: From the Nuclear Interior to the Cytoplasm

Nuclear egress is a process critically required to release nucleocapsids to the cytoplasm for a productive viral replication. For efficient nuclear egress, both NEC proteins have to reach the nucleus to get into contact with the mature nucleocapsid. To this end, the NEC needs to be primarily active at the INM and upon availability of the nucleocapsid. Altogether, this strongly points to a tight regulation of nuclear egress and in particular the NEC activity. 

Based on the combined data of many labs, I would like to present the capsid-escort-model that describes an orchestrated sequence of events for a highly regulated and efficient nuclear egress of herpesviral nucleocapsids: following their cytoplasmic synthesis, pUL31 and pUL34 orthologs take separate and distinct transport routes to the nucleus (Figure 2A,B). Premature interaction of pUL31 and pUL34 already in the cytoplasm is inhibited potentially due to a specific conformation and/or binding partners. Once in the nucleus, pUL31 interacts with nucleocapsids at the sites of assembly and potentially assists in genome packaging and capsid maturation (Figure 2C,D). While the C-terminal domain of pUL31 is sufficient for interaction with nucleocapsids, its N-terminal domain is required for release of nucleocapsid from replication compartments (Figure 2E,F). Capsid-associated pUL31 and membrane-anchored pUL34 are unmasked for formation of heterodimeric NECs that assemble into hexagonal lattices to curve the INM around nucleocapsids until primary envelopment is complete (Figure 2G). NECs or NEC seeds preexisting at the INM could contribute to capsid recruitment and envelopment (Figure 2H,I). Nucleocapsids with primary envelopes temporarily reside in the perinuclear space (Figure 2J) and are de-enveloped and released to the cytoplasm upon fusion of the primary virus envelope with the ONM (Figure 2K). 

A first level of regulation of the NEC is important in the cytoplasm where naturally both proteins co-exist. Evidence is increasing that both pUL34 and pUL31 are regulated to prevent premature interaction. In all NEC structures, pUL31 is characterized by a hook-like extension that embraces pUL34 forming extended interfaces (Figure 4; [38,39,40,41]). Studies on MCMV M50, the pUL34 ortholog, showed that it can adopt two different conformations [118]: if expressed alone, M50 could be masked due to a closed conformation. In the presence of a synthetic peptide that mimics the binding domain of the pUL31 ortholog M53, M50 shows an open conformation with a groove that allows for binding of pUL31. Thus, hydrophobic regions buried in the monomeric conformation become surface exposed in the NEC heterodimer for interaction with pUL31. It is conceivable that the corresponding surface of pUL31 also needs to be masked prior to NEC formation, potentially by proteins other than pUL34 or also by a closed conformation. Interestingly, in presence of the flexible N-terminal domain of pUL31, premature interaction with pUL34 is prevented [17]. Thus, the N-terminal domain could directly or indirectly protect the otherwise exposed V-shaped hook of pUL31. 

In the infected nucleus, two possibilities obviously exist how capsids and NECs could come into contact. The membrane-anchored pUL34 may readily recruit the imported pUL31 to form the NEC and NEC seeds at the INM. Docking of capsids at such NECs pre-existing at the INM is expected to invoke a signal transferred to the MP part resulting in conformational changes, NEC oligomerization and ultimately budding (Figure 2; [68]). Since the NEC can spontaneously nucleate and induce membrane vesiculation [33,43,44], a pre-existing NEC is expected to be kept inactive most likely by other viral proteins. The viral kinase pUS3 represents an interesting candidate for negative regulation, since deletion of the US3 gene results in extensive membrane vesiculation [20,121,124]. US3 however is a nonessential gene and only found in alphaherpesviruses requiring additional regulatory mechanisms [70]. 

Alternatively, as shown by Funk et al., 2015 [17] and recently confirmed by Marschall and Milbradt (personal communication), imported pUL31 can directly target to replication compartments where it efficiently associates with capsids [17]. Consequently, pUL31 is unavailable for NEC formation at the INM. Following release from replication compartments, a process involving the N-terminal domain of pUL31 [17], pUL31 escorts capsids towards the INM-anchored pUL34. There, pUL31 decorating the capsid surface could readily form multiple heterodimeric NECs promoting nucleation of the coat. Positioning of the V-shaped hook of pUL31 may not only permit the embracement of pUL31 and pUL34. It may also bring pUL31-N in close proximity to the MP side of the NEC [38,45]. As shown for HSV1 pUL31, the N-terminal domain and in particular residues 41–50 could interact with the membrane and support vesiculation [45,46]. 

The underlying molecular processes of nuclear egress that most likely involve conformational alterations of the NEC partners are expected to be complex and likely regulated by cellular and viral kinases ([95] and references therein; [127,128,129]) as well as cellular and viral interaction partners [94,104,105,106,107,108]. While undoubtedly, crucial features of the nuclear egress are conserved throughout the herpesviruses, variations on the theme are expected depending on viral strains and cellular hosts.
